# Assessing Colorectal Cancer Screening Knowledge at Tribal Fairs

**Published:** 2010-12-15

**Authors:** Priscilla R. Sanderson, Neil Weinstein, Nicolette Teufel-Shone, María Elena Martínez

**Affiliations:** Northern Arizona University, College of Health and Human Services, Health Sciences Department, College of Social and Behavioral Sciences, Department of Applied Indigenous Studies. At the time of the study, Dr Sanderson was a research associate/postdoctoral fellow with the Cancer Prevention and Control Program, Arizona Cancer Center, College of Medicine, University of Arizona, Tucson, Arizona; University of Arizona, Tucson, Arizona; University of Arizona, Tucson, Arizona; University of Arizona, Tucson, Arizona

## Abstract

**Introduction:**

Increasing public awareness and knowledge about the need for colorectal cancer (CRC) screening among American Indians is key to reducing health disparities. The objective of this study was to assess Navajo adults' knowledge of CRC risk factors and prevention, CRC screening, and self-reported experience with CRC screening.

**Methods:**

We collected data generated by a self-administered survey given to Navajo adults, most of whom lived on the reservation. Data were collected at 2 annual tribal fairs in 2006. Fair attendees who visited an exhibit booth completed a CRC knowledge survey. The study design was nonrandomized.

**Results:**

Of the 285 Navajo adults who participated, most were bilingual (74%) and female (80%). Of the respondents aged 50 years or older, 77% had heard of CRC screening and 28% reported being screened for colon or colorectal cancer. Knowledge was high (mean, 5.78 [standard deviation (SD), 1.28]) (8.0 was the highest possible knowledge score). Respondents with little or no formal schooling had lower scores (mean, 5.4), indicating less knowledge of CRC and associated screening tests than did those with more education (mean, 6.0).

**Conclusion:**

Among a sample of Navajo adults aged 50 years or older, participants with more education were more likely to be knowledgeable about CRC and to have received screening. This survey, led by a Navajo investigator with Navajo surveyors, revealed a high awareness of CRC and screening, but overall CRC screening was low. CRC education for Navajo adults who have little or no formal schooling should be improved.

## Introduction

Colorectal cancer (CRC) is the third leading cause of death for American Indians and Alaska Natives (AI/ANs) (1). In addition, AI/ANs are less likely to be diagnosed at early stages of CRC (59.6%) than non-Hispanic whites (66.5%) (1,2). Wiggins et al reported an age-adjusted incidence rate of colon and rectum cancer of 46/100,000 for Indian Health Service (IHS) Contract Health Service Delivery Area (CHSDA) counties, where misclassification of AI/AN race occurs less frequently than in other areas of the country (3). This rate is lower than that reported for non-Hispanic whites (50.8/100,000) (3). Using data from the Behaviorial Risk Factor Surveillance System (BRFSS), Espey and colleagues reported that AI/ANs aged 50 years or older residing in the southwestern IHS region had a 12% fecal occult blood test (FOBT) uptake within the past year and 21% had endoscopy within the past 5 years (1).

We assessed Navajo adults' knowledge of CRC risk factors and prevention, CRC screening, and self-reported experience with CRC screening. The aims were to determine 1) what proportion of this population had heard about CRC and CRC screening; 2) what proportion of people older than 50 self-reported having been screened; and 3) the relationship between knowledge of CRC and screening by variables such as age, sex, education, and English-language proficiency.

## Methods

The bilingual Navajo surveyors led by the Navajo principal investigator (P.R.S.) recruited Navajo adults who attended the fair exhibits at the 95th Annual Northern Navajo Fair from September 28 through October 1, 2006, and the 38th Annual To'Nanees'Dizi' Fair from October 11 through 15, 2006. We recruited volunteers to complete a brief anonymous assessment of CRC screening knowledge (Appendix). The Navajo respondents were addressed in their preferred language when they approached the booth and spoke either English or Navajo to the Navajo research team.

We based survey development on the health belief model's constructs of perceived susceptibility and perceived benefits of prevention (4), the literature on brief health surveys with underserved populations (5-7), and the National Cancer Institute web page on CRC screening. Questions were built on those used previously in surveys of CRC screening (8-11) with recommendations from the Navajo project advisory committee.

A Navajo project advisory committee composed of representatives from the Navajo Division of Health's Navajo Breast and Cervical Cancer Project, Community Health Representatives (CHRs), and Northern Navajo Medical Center's Health Promotion Program assisted with cultural relevancy in the survey. The primary author (P.R.S.) met several times with the Navajo project advisory committee for feedback on the survey drafts. This committee of 6 members recommended adding the following items: 1) a question on general health, 2) a "don't know" option on the multiple choice questions, 3) age-group choices, and 4) an open-ended highest-education question that allowed the respondents to write in the highest level of education attained.

The 16-item survey included a multiple choice question about general health, a yes or no question about "ever heard" the terms "colon cancer or colorectal cancer," and a self-report question about experience with CRC. Five questions addressed perceived susceptibility to CRC related to sex, age, family history, and lifestyle; 2 questions asked about prevention; 1 about symptoms; and 5 about demographic status (sex, tribal affiliation, age group, highest level of education attained, and language spoken on a daily basis).

Respondents self-administered the paper-and-pencil questionnaire, which was written in English. We administered the questionnaire orally in Navajo if requested.

The University of Arizona Human Subjects Protection Program, Navajo Area Indian Health Boards, the Navajo Nation Agency Councils, and the Navajo Nation Human Research and Review Board approved the project. The primary author (P.R.S.) and the project advisory committee received approvals from 4 Navajo Nation Agency Councils (Northern, Western, Fort Defiance, Chinle), each of which required a tribal council resolution. The process of gathering these approvals took more than 1 year; the proposed research protocol was then submitted to the Navajo Nation Human Research and Review Board for approval.

We conducted an analysis to determine whether the number of correct answers to the 8 knowledge questions could be considered a reliable measure of screening knowledge. For this scale, Cronbach α, a measure of internal consistency, was low at .50, indicating that the total knowledge score should be interpreted cautiously and that individual knowledge questions should be examined individually. Because the relationship between CRC rates and sex is complex, we excluded from the total knowledge score the answer to "Men have a much higher risk for colon cancer than women."

We scored the survey on the basis of shared (correct) responses to the 8 true/false knowledge questions. We used SPSS 15.0 for Windows (SPSS, Inc, Chicago, Illinois) for data analysis. To determine the relationships between having heard of colon cancer and being screened and demographic characteristics, we conducted χ^2^ analyses in addition to simple frequencies. We used *t* tests and analysis of variance tests to determine whether the total knowledge scores were related to other variables. Unless otherwise indicated, statistical tests were 2-sided and *P* values of less than .05 were considered significant.

## Results

Of the 285 Navajo respondents who completed the questionnaire, 39% were aged 50 or older (Table 1). Approximately half of the women (52%) were aged 30 to 49 years; most of the men (49%) were aged 50 to 79 years. Education ranged from no formal education to completion of a doctoral degree.

### Knowledge of risk factors and prevention

The entire sample had an error rate of less than 20% on the knowledge statements (21 missing and "don't know"  responses were not counted as shared responses) (Table 2). The mean score on the 7-item knowledge scale was 5.78 (standard deviation [SD], 1.28).

We examined the relationship between knowledge and screening by comparing the knowledge scores of those aged 50 or older who were screened and those who were not screened (Table 3). Respondents who reported having been screened had a higher degree of accuracy on all questions. The *t* test on the total knowledge score (based on 7 questions) indicated that respondents who were screened (mean, 6.2) had a significantly higher knowledge score than those who were not screened (mean, 5.4; *P* = .001).

### Relationship between sociodemographic variables, CRC, and CRC screening knowledge

Seventy-four percent of respondents who were aged 49 or younger reported having heard of CRC, compared with 77% for those aged 50 or older. More than half of both women (77%) and men (65%) reported having heard of CRC (*P* = .032).

Level of education was a factor in knowledge of CRC; 88% of respondents with more than a high school education had heard about CRC, compared with 42% of those who had a high school education or less (*P* = .001). People who said they had heard of CRC were significantly more knowledgeable about prevention and risk (had 6 correct answers to the 8 true/false questions) than those who said they had not.

CRC screening knowledge was not significantly related to sex, age group (Table 3), or self-reported health (Table 4). Respondents who spoke only English (n = 44) had higher scores than those who spoke only Navajo (n = 29) or were bilingual (n = 212).

### CRC screening

Screening status was related to sex, education, language, and health status (Table 5). "Don't know" responses were counted as not screened for CRC. Respondents who had heard about CRC screening were significantly more likely to have been screened (7% of respondents aged 49 years or younger and 28% of those aged 50 or older reported having been screened for CRC).

Most respondents reported having had their CRC screening test at an IHS hospital on the Navajo Reservation. Most respondents (54%) were screened in Arizona and the remainder in New Mexico (37%), Colorado (7%), or Utah (2%). We asked no further questions related to CRC screening experience. Screening was not significantly related to the rest of the variables.

## Discussion

Incidence and mortality rates of CRC among AI/ANs vary widely by region; AIs in the Southwest have the lowest rates (1). Furthermore, AI/ANs are less likely to receive a diagnosis of early-stage CRC than non-Hispanic whites, and this difference is greatest for people who live in the Southwest (1). A later stage at diagnosis could result from lack of screening. Using data from the BRFSS, Espey et al show that although AI/ANs have lower rates of CRC screening (FOBT or endoscopy) than non-Hispanic whites, the difference is most pronounced for people in the Southwest (1).

Data on knowledge of CRC prevention and screening among AI/ANs are sparse. To fill this gap, we collected data on knowledge of CRC risk factors and prevention and CRC screening among Navajo adults. Because the Navajo population is widely dispersed on the Navajo Reservation, which covers New Mexico, Arizona, and Utah, access to this group is challenging. Obstacles include poor road conditions, a low percentage of landline telephones, a lack of transportation, and language barriers. A potentially effective way to reach Navajo adults is by meeting them at the annual Navajo Nation fairs.

The American Cancer Society identifies risk factors for developing possible colorectal polyps or CRC as age, especially being aged 50 or older; a personal or family history of CRC; inflammatory bowel disease, such as Crohn disease or ulcerative colitis; and inherited genetic diseases, such as familial adenomatous polyposis and hereditary nonpolyposis colorectal cancer, and type 2 diabetes; and certain lifestyle factors (12). The lifestyle factors include lack of exercise; a high-fat, low-fiber diet; obesity; smoking; and high intake of alcoholic beverages (13).

The development of this survey, which was implemented with input from local tribal community representatives, was similar to that of Guadagnolo et al (13). This input and involvement is essential in Indian country. The primary author consulted with the study's project advisory committee for input in survey development to show courtesy and respect and to draw on their expertise in working with Navajo adults. This input addressed the unique geography, language, practices, beliefs, and attitudes characteristic of many rural tribal communities.

Results of our work are supported by those of Tseng et al, in which Navajo adults aged 50 or older who had heard of and had been screened for CRC were more knowledgeable than those who were not screened (14). Our results also show that knowledge of CRC screening did not vary by sex (14).

More than half of Navajo women and less than half of Navajo men had heard of CRC. The significance of this finding may be linked to the Education and Research Towards Health (EARTH) study conducted March 2004 through July 30, 2007 (15). Our study was conducted from September through October 2006 when the EARTH study was in progress on the Navajo reservation. The EARTH study recorded self-reported screening rates for cervical, breast, and colorectal cancer. The EARTH study (15) results show that higher education, higher income, and having 1 or 2 medical conditions are associated with cancer screening, in accordance with the outcome of our survey.

Some limitations of this study warrant consideration. The first is that all data are self-reported. People may have exaggerated their awareness of CRC or the frequency with which they have been screened. Some may have confused a digital rectal exam with CRC screening, which would inflate the screening rates. A second limitation is that the respondents were not a random sample of Navajos. People who attend tribal fairs are unlikely to be representative of all members of the Navajo Nation, and people who approached the Arizona Cancer Center (AZCC) booth may have been more interested in and knowledgeable about cancer issues than others at the fairs who did not approach the AZCC booth or table. Thus, the screening rate observed here should not be used as a rate for the Navajo Nation as a whole. A third limitation is that the data were cross-sectional. We cannot comment on whether knowledge tends to increase screening or whether screening tends to increase knowledge.

Few Navajo men participated in this study. We had one male Navajo surveyor in the same booth with the primary author and a female Navajo surveyor. For future research, we suggest having separate fair survey booths (male Navajo investigators to survey Navajo men and female Navajo investigators to survey Navajo women). This change may encourage Navajo men aged 50 or older to complete a CRC knowledge survey. Besides being near the tribal exhibit booths, the survey booths could be set up close to the rodeo grounds and song-and-dance arena; most Navajo men aged 50 or older participate in or watch these events.

Results of our study show that knowledge of CRC screening among Navajo adults was high but that overall screening was low. In addition, people with higher education were more likely to have heard of CRC and to have received screening. These results support the need to develop culturally appropriate information in both the English and Navajo languages, specifically tailored to those who are aged 50 years or older. This tailored screening information needs to take into account low literacy levels and may need to consider oral and written methods.

## Figures and Tables

**Table 1:** Demographic Characteristics of Navajo Adults (N = 285), Colorectal Cancer Screening Knowledge Survey, September-October 2006

**Characteristics**	**No. (%)**
**Age,^a^ y**	
18-29	38 (13)
30-39	58 (20)
40-49	79 (28)
50-59	73 (26)
60-79	36 (13)
**Sex^a^**	
Men	55 (19)
Women	228 (80)
**Education**	
No education (no formal schooling) through 8th grade	16 (6)
9th grade through completion of 12th grade or completion of GED	104 (36)
13 (associate degree or trade school)	29 (10)
14 (some college)	68 (24)
Bachelor's or graduate-level degree	68 (24)
**Daily language spoken at home**	
Navajo	29 (10)
English	44 (16)
Both Navajo and English	212 (74)
**Health status^a^**	
Excellent	37 (13)
Very good	91 (32)
Good	113 (40)
Fair	36 (13)
Poor	7 (3)

Abbreviation: GED, General Educational Development certificate.

a Nonresponses for age and sex not counted.

**Table 2:** Frequency of Correct Answers, by Entire Sample and Navajo Sample Aged 50 Years or Older Who Were Screened or Not Screened for Colorectal Cancer, Colorectal Cancer Screening Knowledge Survey, September-October 2006

Knowledge Statements Followed by Correct Answers (T) or (F)	Respondents Who Answered Each Question, No.	Entire Navajo Sample Aged 18-79, No. (%) n = 285	Navajo Aged ≥50 Screened, No. (%) n = 30	Navajo Aged ≥50 Not Screened, No. (%) n = 78
1. Colon cancer can almost always be prevented or detected early with screening tests. (T)	281	253 (90)	30 (97)	64 (85)
2. Simple lifestyle changes, such as improving your diet and increasing physical activity, can reduce your risk of getting colon cancer. (T)	281	241 (86)	25 (83)	64 (84)
3. People cannot get colon cancer unless it runs in their family. (F)	279	234 (85)	26 (87)	55 (75)
4. People can have colon cancer without having any symptoms. (T)	284	239 (84)	28 (90)	62 (82)
5. A colon cancer screening test is not a one-time event. Beginning at age 50, average-risk people should be screened on a regular basis. (T)	282	235 (83)	29 (93)	65 (85)
6. Once you get colon cancer, there isn't anything you can do about it. (F)	282	216 (77)	22 (73)	53 (70)
7. Men have a much higher risk for colon cancer than women. (T)	275	181 (66)	22 (71)	48 (66)
8. All people's risk for developing colon cancer increases as they age. (T)	281	223 (79)	27 (90)	57 (76)

**Table 3:** Total Knowledge Scores of Participants (N = 285) in a Colorectal Cancer Screening Knowledge Survey, September-October 2006

Characteristic	Total Sample^a^	Knowledge Score^b^	Standard Deviation	*t* Score	*df*	*P* Value^c^
Men	55	5.7	1.42	.192	77.3	.85
Women	228	5.8	1.30			
**Education**						
Less education (grades 0-12: no formal education to completion of 12th grade)	120	5.4	1.43	3.51	226	.001
More education (grades 13-18: associate of arts degree, trade school, some college, bachelor's or graduate-level degree)	165	6.0	1.18			
Aged ≤49 y	175	5.8	1.24	1.24	208	.22
Aged ≥50 y	109	5.6	1.40			
Heard of CRC	214	6.0	1.06	5.20	90.2	<.001
Not heard of CRC	71	5.0	1.65			
Screened for CRC and aged ≥50 y	30	6.2	0.93	3.30	84.6	.001
Not screened for CRC and aged ≥50 y	78	5.4	1.50			

Abbreviations: *df*, degrees of freedom; CRC, colorectal cancer.

a Nonresponses not counted.

b Correct answers out of 7.

c Calculated by χ^2^ test.

**Table 4:** Language Spoken and Health Characteristics of Participants (N = 285) in a Colorectal Cancer Screening Knowledge Survey, September-October 2006

Characteristic	**Total Sample (Mean), ** a No.	Knowledge Score (SD)^b^	*F*	*df*	*P* Value^c^

**Daily language spoken at home**					
Navajo	29	5 (1.24)	5.94	2,282	.003
English	44	6 (0.89)			
Both Navajo and English	212	6 (1.37)			
**General health**					
Excellent	37	6 (1.47)	1.45	4,279	.22
Very good	91	6 (1.15)			
Good	113	6 (1.39)			
Fair	36	6 (1.19)			
Poor	7	5 (1.67)			

Abbreviations: *F*, variance ratio distribution; *df*, degrees of freedom.

a Nonresponses not counted.

b Correct answers out of 7.

c Calculated by one-way analysis of variance test.

**Table 5:** Demographic Characteristics of Participants Aged 50 Years of Age or Older, by Screening Status, in a Colorectal Cancer Screening Knowledge Survey, September-October 2006

Characteristic	Screened for CRC,No. (%)	Not Screened for CRC,No. (%)	Total^a^	*P* Value^b^
Men^c^	8 (30)	19 (70)	27	.51
Women	22 (27)	58 (72)	80	
Total	30 (28)	77 (72)	107	
**Education**				
(0-12: no formal education to completion of 12th grade)	10 (20)	40 (80)	50	.07
More education (grades 13-18: associate's degree, trade school, some college, bachelor's or graduate-level degree)	20 (34)	38 (65)	58	
**Daily language spoken at home**				
Navajo	5 (29)	12 (71)	17	.67
English	0	2 (100)	2	
Both Navajo and English	25 (28)	64 (71)	89	
**General health^c^**				
Excellent	3 (19)	13 (81)	16	.90
Very good	8 (27)	22 (73)	30	
Good	14 (32)	30 (68)	44	
Fair	4 (30)	9 (70)	13	
Poor	1 (25)	3 (75)	4	
**Ever heard of CRC screening**	28 (33)	57 (67)	85	.047
Not heard of CRC screening	2 (9)	21 (91)	23	

Abbreviations: CRC, colorectal cancer.

a Nonresponses not counted.

b Calculated by using *t *test.

c One respondent did not answer the question.

## Appendix. Anonymous Assessment

Check one answer:

1. Would you say that in general your health is:

Excellent........□ Very good........□ Good........□ Fair........□ Poor........□

Please answer the questions below (circle one).

2. Have you ever heard of "colon cancer" or "colorectal cancer"? ........ **Yes** or **No**

3. Have you had a colon cancer screening test? ........ **Yes** or **No** or **Don't Know**

If yes, when? _____________________________________________________ Where? __________________________________________________________

Go to page 2 →

Please indicate whether you think each of the following statements is true or false (circle one).

4. People can have colon cancer without having any symptoms........**True or False**

5. Men have a much higher risk for colon cancer than women........**True or False**

6. A colon cancer screening test is not a one-time event.

Beginning at age 50, average-risk individuals should be screened on a regular basis.........**True or False**

7. Colon cancer can almost always be prevented or detected early with screening tests.........**True or False**

8. People cannot get colon cancer unless it runs in their family.........**True or False**

9. Simple lifestyle changes, such as improving your diet and increasing physical activity, can reduce your risk of getting colon cancer.........**True or False**

10. All people's risk for developing colon cancer increases as they age.........**True or False**

11. Once you get colon cancer, there isn't anything you can do about it.........**True or False**

Go to page 3 →

Gender (circle one): **Male or Female**

Navajo (circle one): **Yes or No**

If no, what is your race/ethnicity? _____________________________________

My age group is (check one):

18 – 20........□ 21 – 29........□ 30 – 39........□ 40 – 49........□ 50 – 59........□ 60 – 69........□ 70 – 79........□ 80 – 89........□ 90 & over......□

Your highest education is: _____________________________

On a daily basis, do you speak (check one):

Navajo........□ English........□ Both Navajo and English........□ Other______________________________

Thank you for taking time from your busy day to complete this assessment.

Enjoy the fair!
